# Supplementary material for: Brain-wide visual habituation networks in wild type and fmr1 zebrafish
Source: Nat Commun. 2022 Feb 16;13:895. doi: 10.1038/s41467-022-28299-4 (PMC8850451; doi:10.1038/s41467-022-28299-4)
Supplement: Supplementary file 1 — Supplementary Information [file 41467_2022_28299_MOESM1_ESM.pdf]

Supplementary Information for

Brain-wide visual habituation networks in wild type and *fmr1* zebrafish

Emmanuel Marquez-Legorreta, Lena Constantin, Marielle Piber, Itia A. Favre-Bulle, Michael A. Taylor, Ann S. Blevins, Jean Giacomotto, Dani S. Bassett, Gilles C. Vanwalleghem, and Ethan K. Scott

Correspondence to: ethan.scott@uq.edu.au

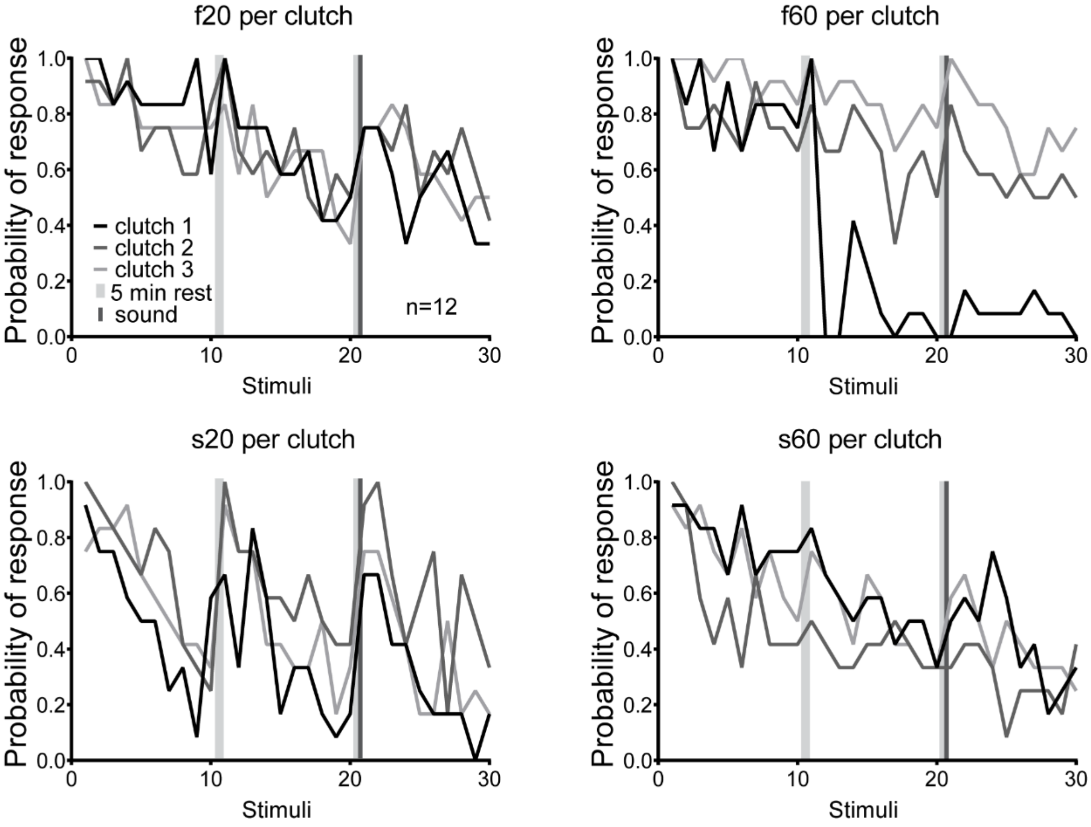

Supplementary Figure 1. Clutch variability during habituation.

Probability of response across 3 different clutches of each of the 4 groups during three blocks of ten loom presentations. Each clutch had 12 fish.

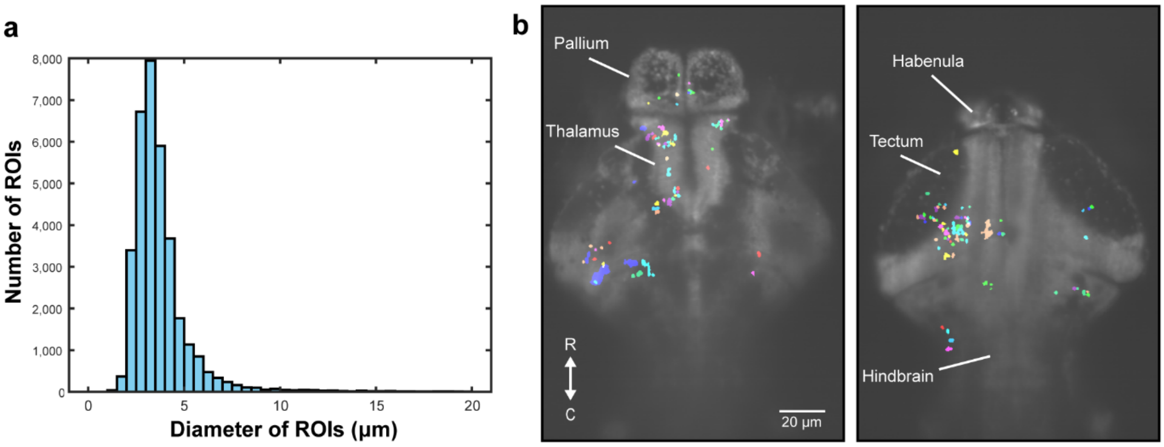

Supplementary Figure 2. Size of ROIs and sample of ROI segmentation

a. Histogram of diameter of loom responsive ROIs from the f20 group. The number of pixels forming each ROIs was extracted from the CaImAn outputs and, assuming circular shapes, the diameter was calculated and transformed to µm. Each pixel is equivalent to 1.2904 µm. **b**. Example slices of a single fish and their loom responsive ROIs.

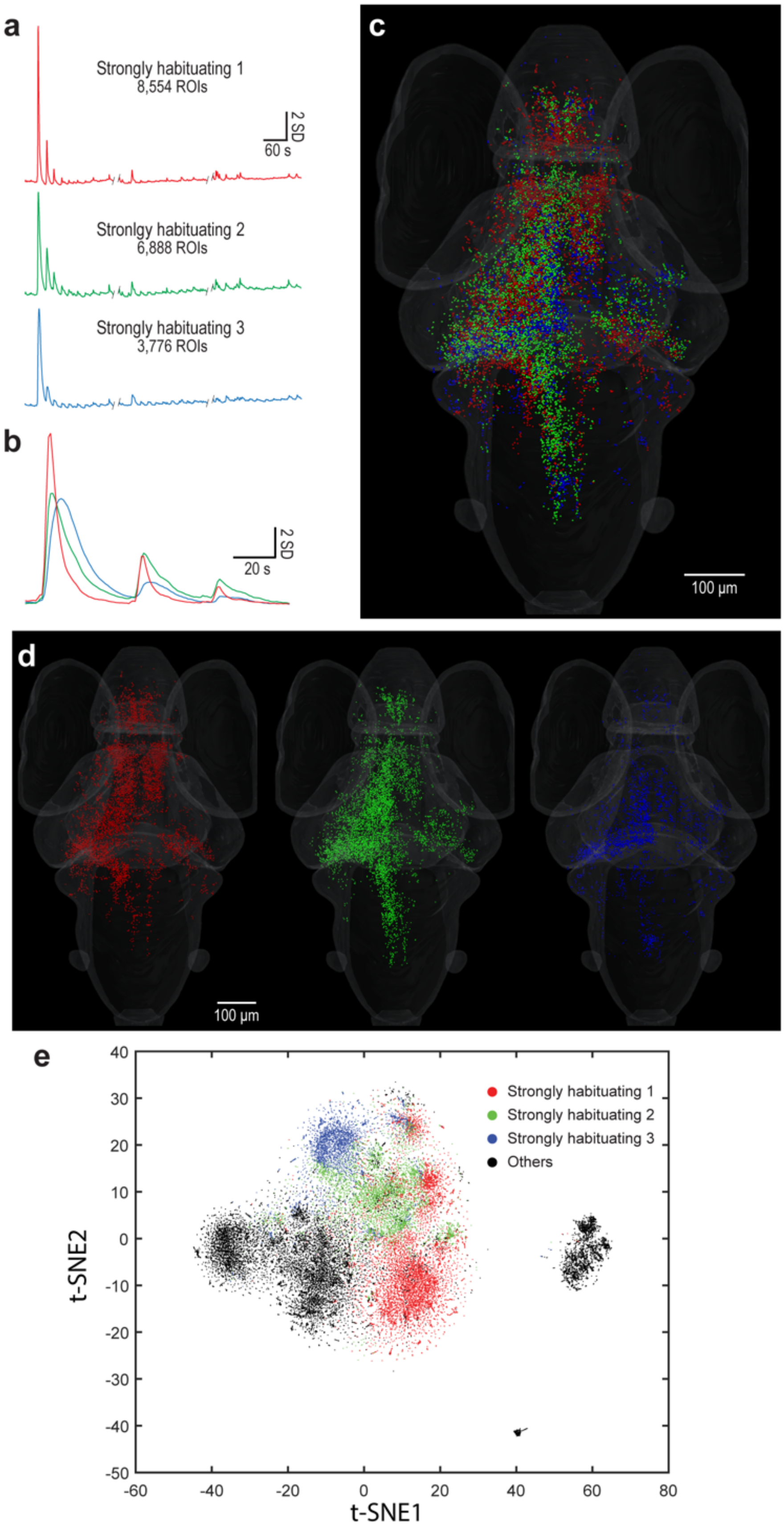

a
Strongly habituating 1
8,554 ROIs
2 SD
60 s
Stronlgy habituating 2
6,888 ROIs
Strongly habituating 3
3,776 ROIs
b
2 SD
20 s
c
100 µm
d
100 µm
e
Strongly habituating 1
Strongly habituating 2
Strongly habituating 3
Others
t-SNE1
t-SNE2

Supplementary Figure 3.

a. The average responses of the ROIs composing three of the clusters produced by the k-means clustering technique. All are powerfully responsive to the first loom stimulus, with strongly attenuated responses in the second and third trials (using the f20 stimulus train). Responses are essentially absent from trials 4-30 across three blocks of ten stimuli. **b**. The response strengths and temporal dynamics are similar across these three groups during the first three trials, although the fast habituating cluster #1 shows the sharpest response profile. **c**. The anatomical locations of these ROIs are indicated, showing a considerable degree of overlap in their distributions (with clusters shown individually in panels **d**). Finally, a t-SNE analysis (**e**) fails to reveal clear functional distinctions among these groups, with extensive overlap and intermingling across these three clusters, especially for the Fast Habituating 2 cluster (green). These functional and anatomical analyses form the basis for our pooling these three groups into a single "strongly habituating" cluster in our subsequent analyses.

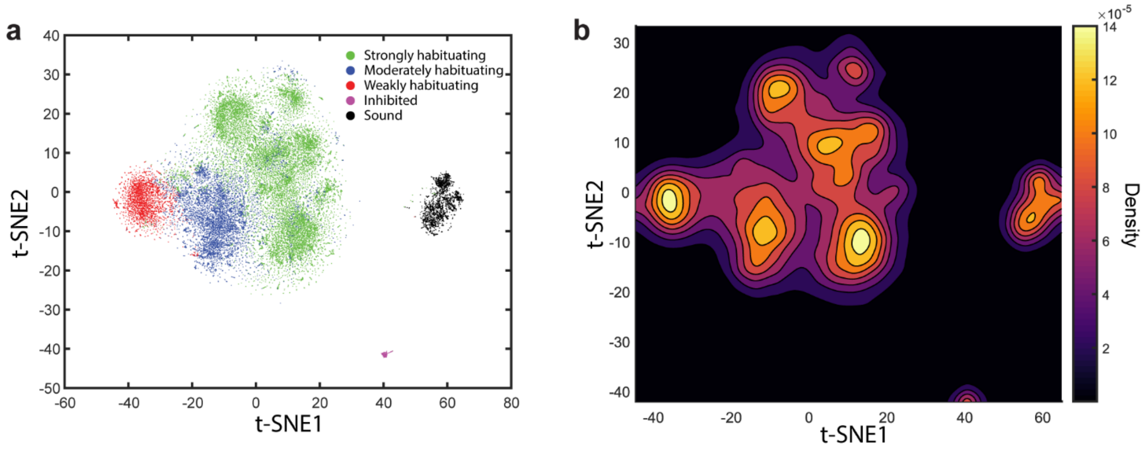

Supplementary Figure 4.

a. The calculated distributions of ROIs belonging to five functional clusters are represented following a t-SNE analysis. A very small overlap is seen across these clusters. The motor associated cluster was not included because the ROIs in this cluster show different patterns of activity in different fish. **b**. Density plot of the t-SNE in **a**.

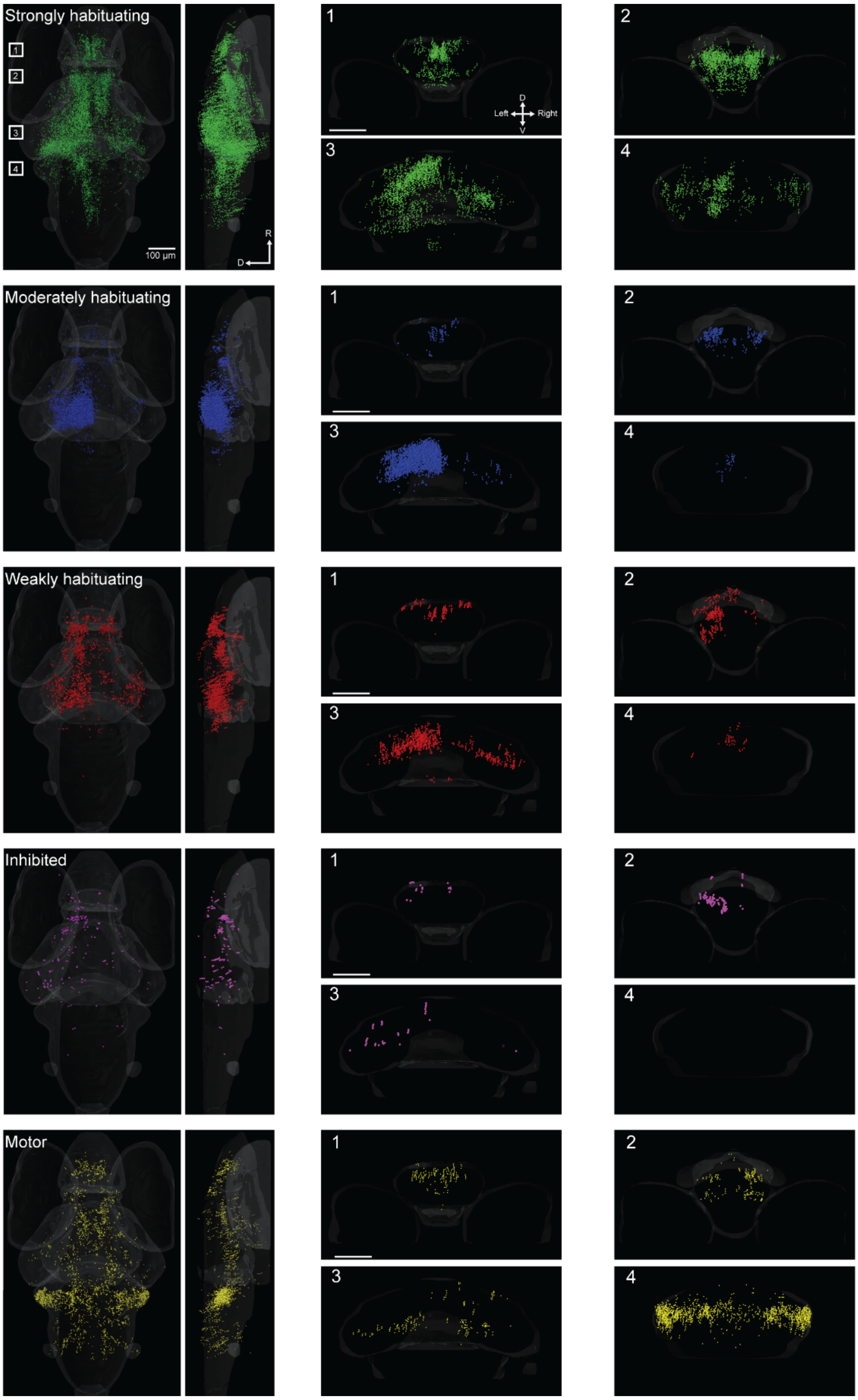

Supplementary Figure 5. Anatomical distributions of five habituating clusters.
For each functional cluster shown in Figure 2, a dorsal view, lateral view, and four coronal virtual sections are shown. The ranges included in the coronal sections are indicated by the height of the corresponding boxes in the top left panel.

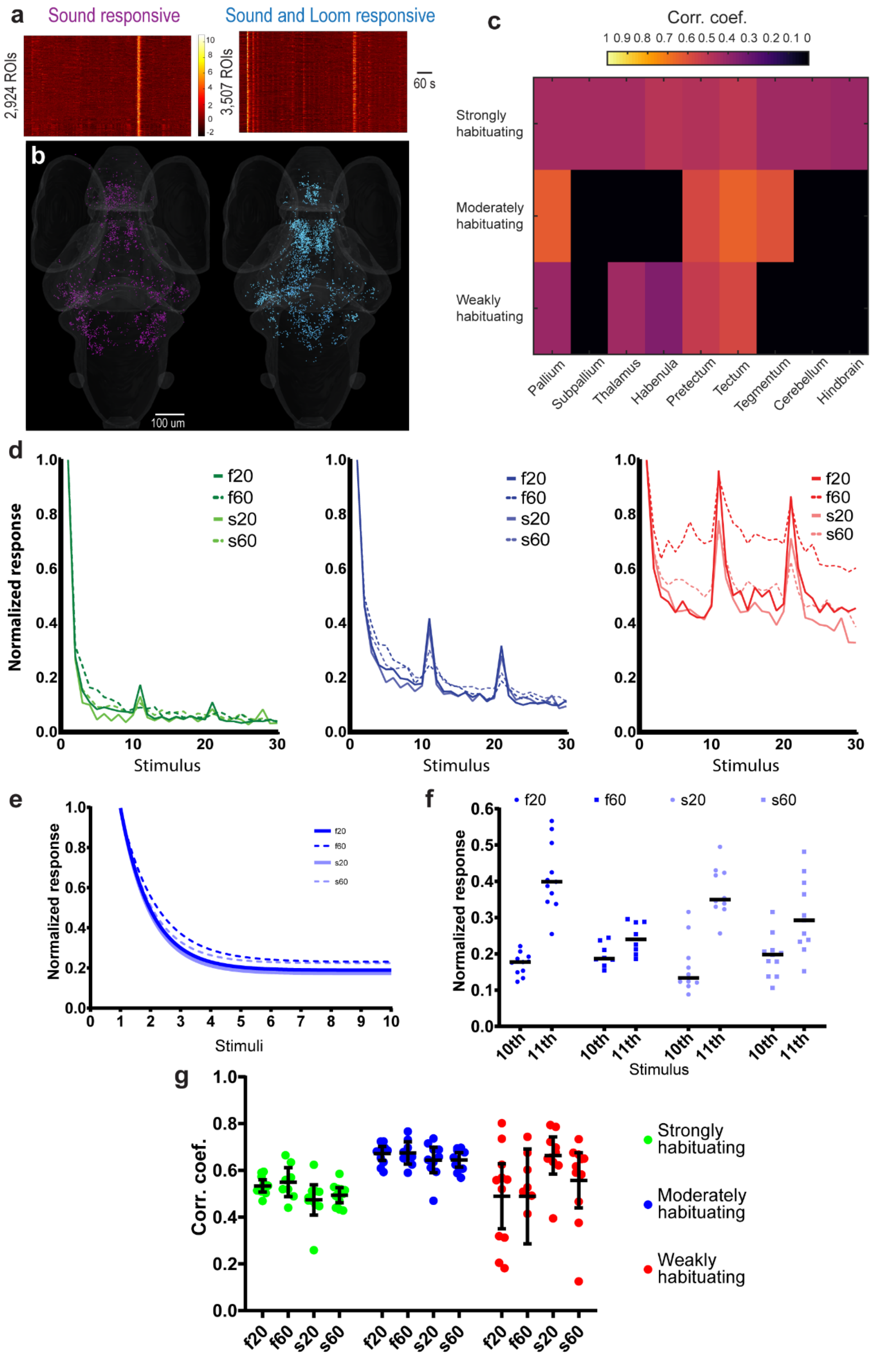
a
Sound responsive
Sound and Loom responsive
2,924 ROIs
3,507 ROIs
60 s
b
100 um
c
Corr. coef.
1 0.9 0.8 0.7 0.6 0.5 0.4 0.3 0.2 0.1 0
Strongly habituating
Moderately habituating
Weakly habituating
Pallium
Subpallium
Thalamus
Habenula
Pretectum
Tectum
Tegmentum
Cerebellum
Hindbrain
d
Normalized response
Stimulus
f20
f60
s20
s60
e
Normalized response
Stimuli
f
Normalized response
10th
11th
Stimulus
g
Corr. coef.
Strongly habituating
Moderately habituating
Weakly habituating

Supplementary Figure 6: Correlation of habituation dynamics between free-swimming and brain responses.

a. Raster plots of ROIs that responded to the auditory stimuli (left) and to both loom and sounds (right). Color bar represents the SD. **b**. Top view of the anatomical locations of the ROIs in **a**. Left for the sound only responsive ROIs (in purple) and right for the loom and sound responsive ROIs (in cyan). **c**. Correlation analysis of habituation dynamics for the free-swimming behaviour and the habituating responses in eight brain regions. **d**. The average response profiles for strongly habituating (left) moderately habituating (center), and weakly habituating (right) tectal ROIs in each of the habituation paradigms. **e**. Fitted curves to the Moderately habituating tectal normalized responses during the first block of habituation. **f**. Recovery responses after first block of stimuli of the Moderately habituating tectal normalized responses. Black horizontal bar represents the median. **g**. Pearson correlations between the normalized responses of the tectum and their group-matched free-swimming probability of responses. Error bars show mean and 95% CI.

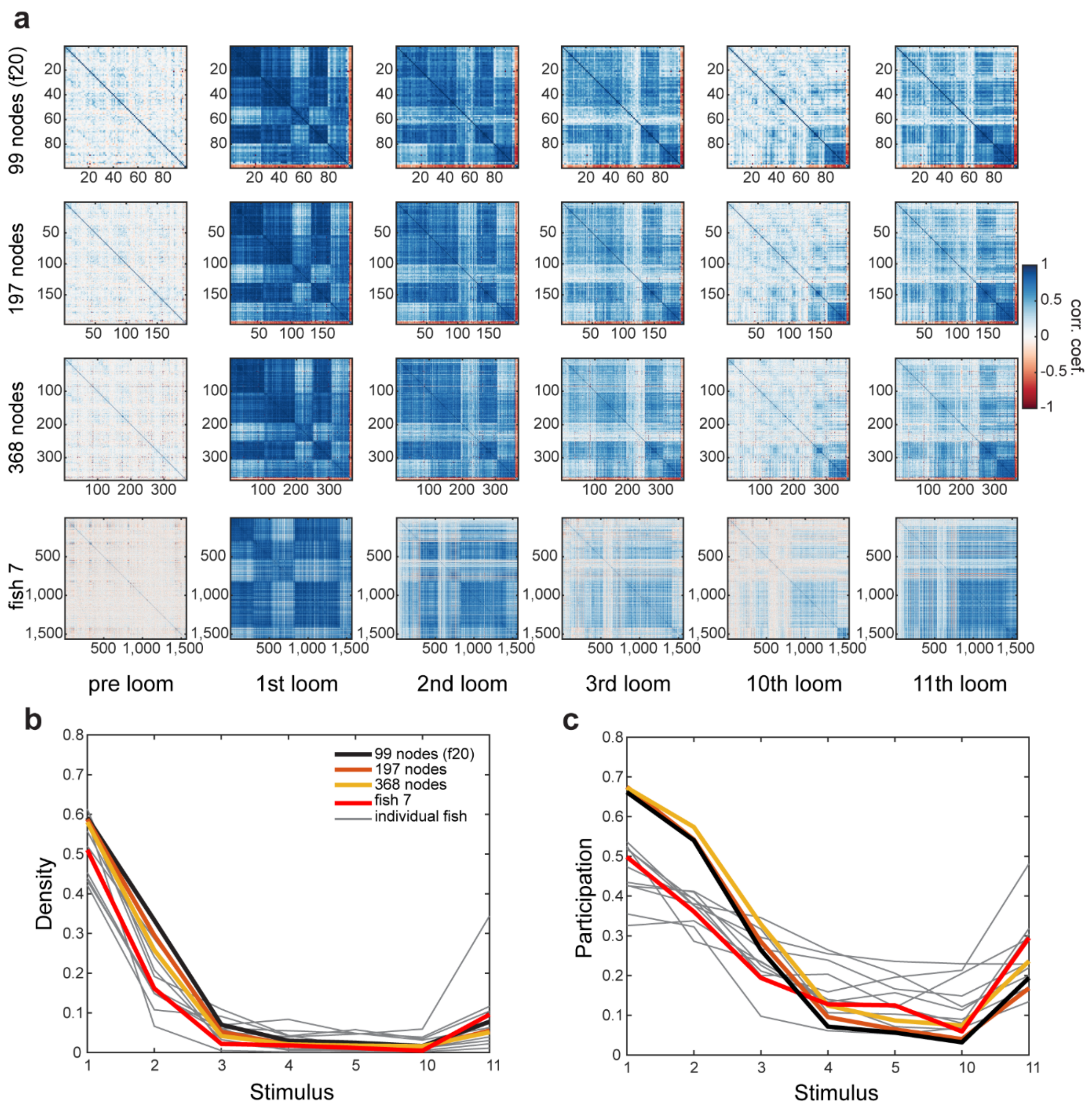

Supplementary Figure 7. Validation of graph theory approach: Number of nodes and single ROI graph analysis.

a. Matrices generated with a scalable number of nodes (99 nodes, first row; 197 nodes, second row; and 368 nodes, third row) and all loom responsive ROIs of a sample fish (fourth row) for the pre-loom moment and the 1st, 2nd, 3rd, 10th, and 11th looms. **b.** Density indices of pair-wise node connections from panel **a** (colored lines) and the all-ROI matrices of eleven individual fish from the f20 group (grey lines). **c.** Participation coefficients of the same graphs as in panel **b** (color labelling is the same).

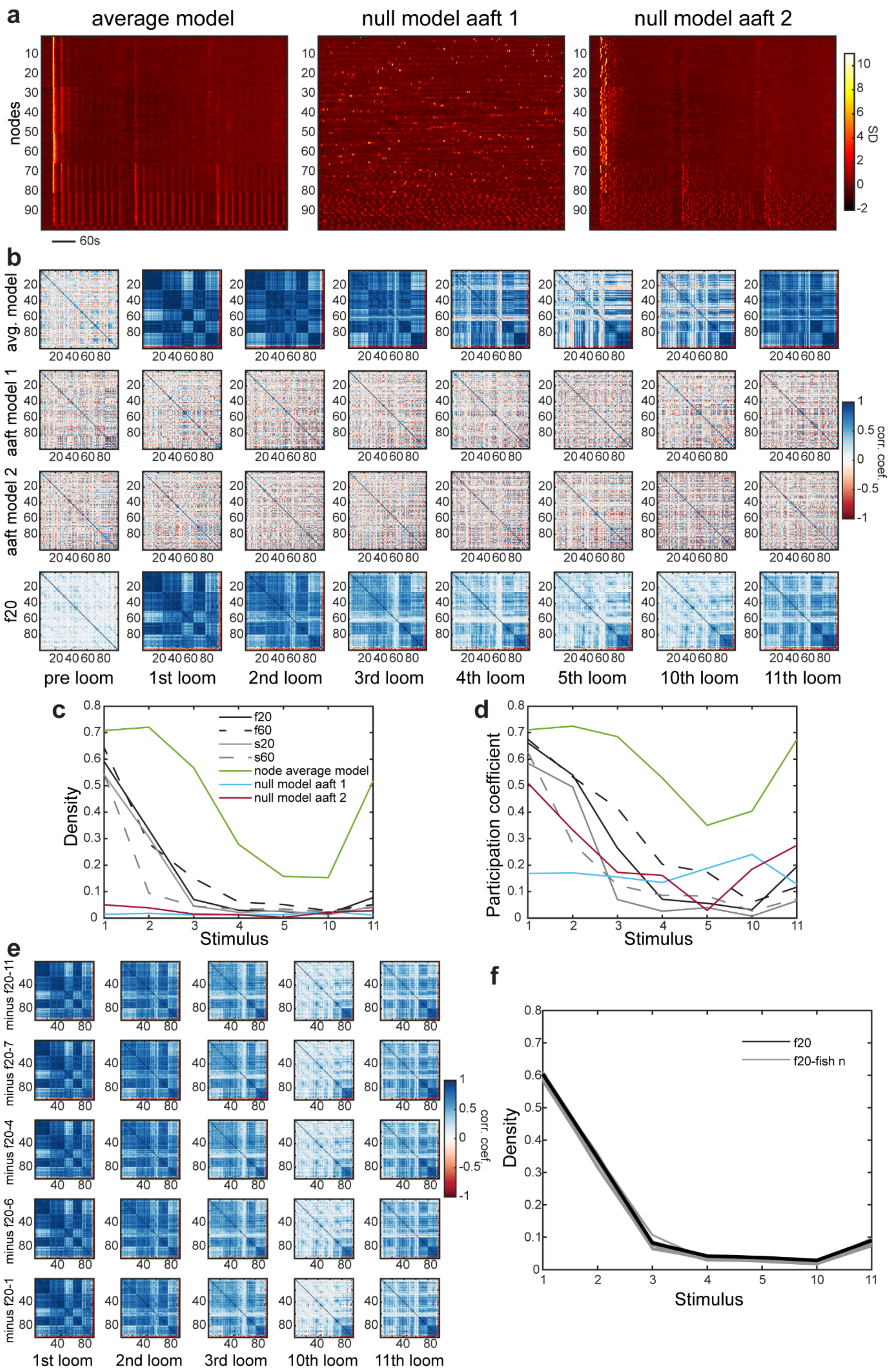

a
average model
null model aaft 1
null model aaft 2
nodes
60s
SD
b
avg. model
aaft model 1
aaft model 2
f20
corr. coef.
pre loom
1st loom
2nd loom
3rd loom
4th loom
5th loom
10th loom
11th loom
c
Density
Stimulus
f20
f60
s20
s60
node average model
null model aaft 1
null model aaft 2
d
Participation coefficient
Stimulus
e
minus f20-11
minus f20-7
minus f20-4
minus f20-6
minus f20-1
corr. coef.
1st loom
2nd loom
3rd loom
10th loom
11th loom
f
f20
f20-fish n
Density
Stimulus

Supplementary Figure 8. Validation of graph theory approach: Null models and leave-one-out cross validation.

a. Raster plots of the nodes' activity for each of the generated models. Left: average activity of f20 fish nodes. Middle: adjusted-amplitude Fourier transform surrogate time series from the average model. Right: adjusted-amplitude Fourier transform surrogate time series from the average model where the AAFT was applied only to the loom presentation windows. **b.** Matrices generated with the models (first to third row) and the f20 dataset (fourth row) for the pre-loom moment, 1st-5th, 10th, and 11th looms. **c**. Density index of the connections of the matrices in panel **b** (colored lines) and the four stimulus train graphs (f20, f60, s20 and s60). **d.** Participation coefficient of the same graphs as in panel **c**. **e.** Sample sets each composed of five matrices generated with the leave-one-out cross-validation method for the pre-loom moment, 1st-3rd, 10th, and 11th looms (the fish excluded from each row is indicated at the left). **f.** Density index of the connections of the leave-one-out generated group-average matrices (grey lines, n=11) and the f20 whole group average matrix (black line) for the 1st-3rd, 10th, and 11th looms.

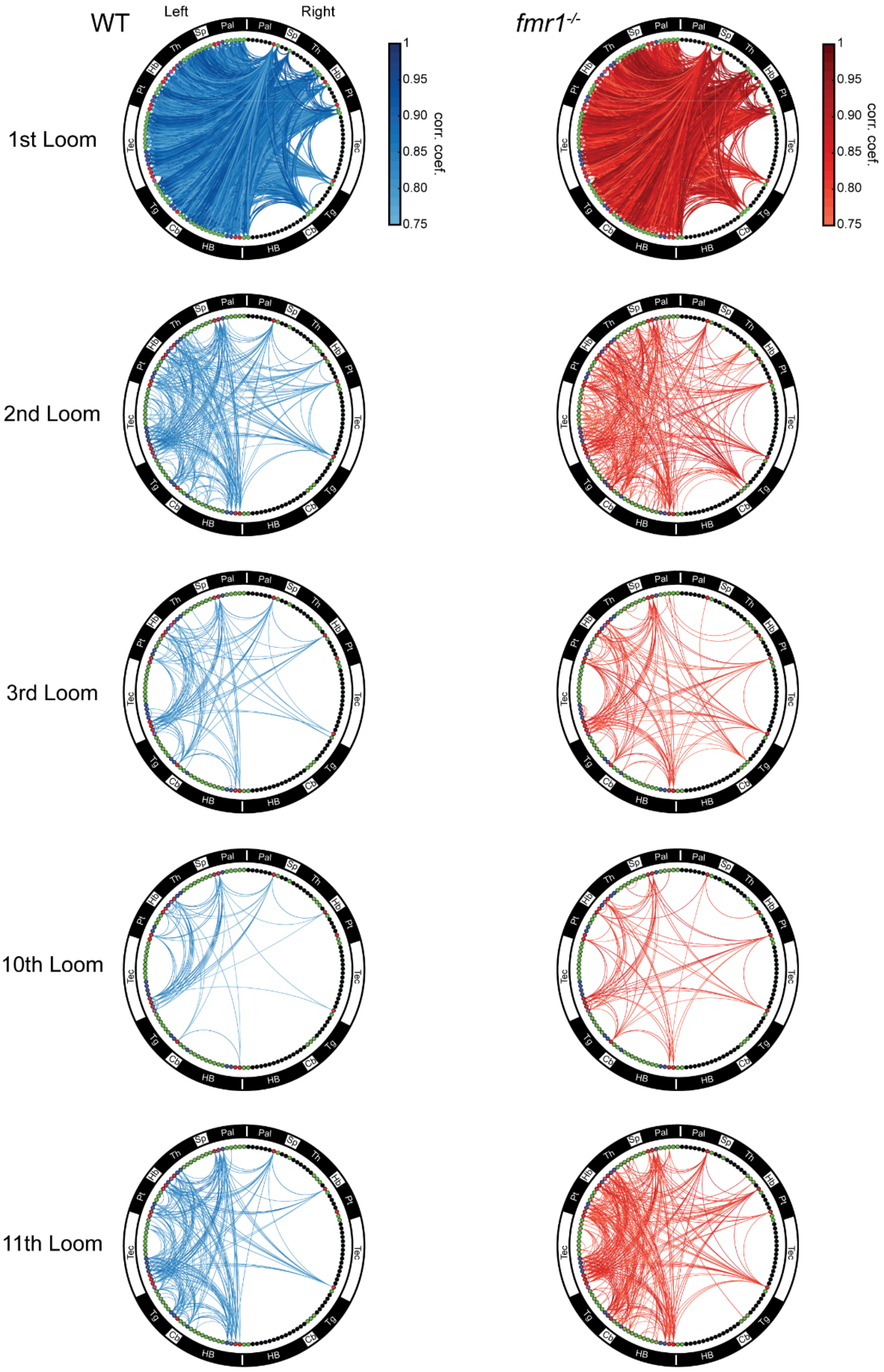

WT
Left
Right
fmr1-/-
1st Loom
2nd Loom
3rd Loom
10th Loom
11th Loom
corr. coef.
1
0.95
0.90
0.85
0.80
0.75
Pal
Sp
Th
Hb
Pt
Tec
Tg
Cb
HB

Supplementary Figure 9. Spatially sorted brain-wide graphs for WT and *fmr1*$^{-/-}$ larvae. Edges with strengths above 0.75 are shown between all pairs of nodes for trials 1, 2, 3, 10, and 11; nodes are arranged by brain region. The nodes' functional clusters are identified by color. Empty nodes (black) are added to spatially match the right side (ipsilateral to the visual stimulus) to the left side, despite the latter having fewer nodes. Abbreviations are the same as in Figure 6: Pallium, Pal; subpallium, Sp; thalamus, Th; habenula, Hb; pretectum, Pt; tectum, Tec; tegmentum, Tg; cerebellum, Cb; and hindbrain, HB.

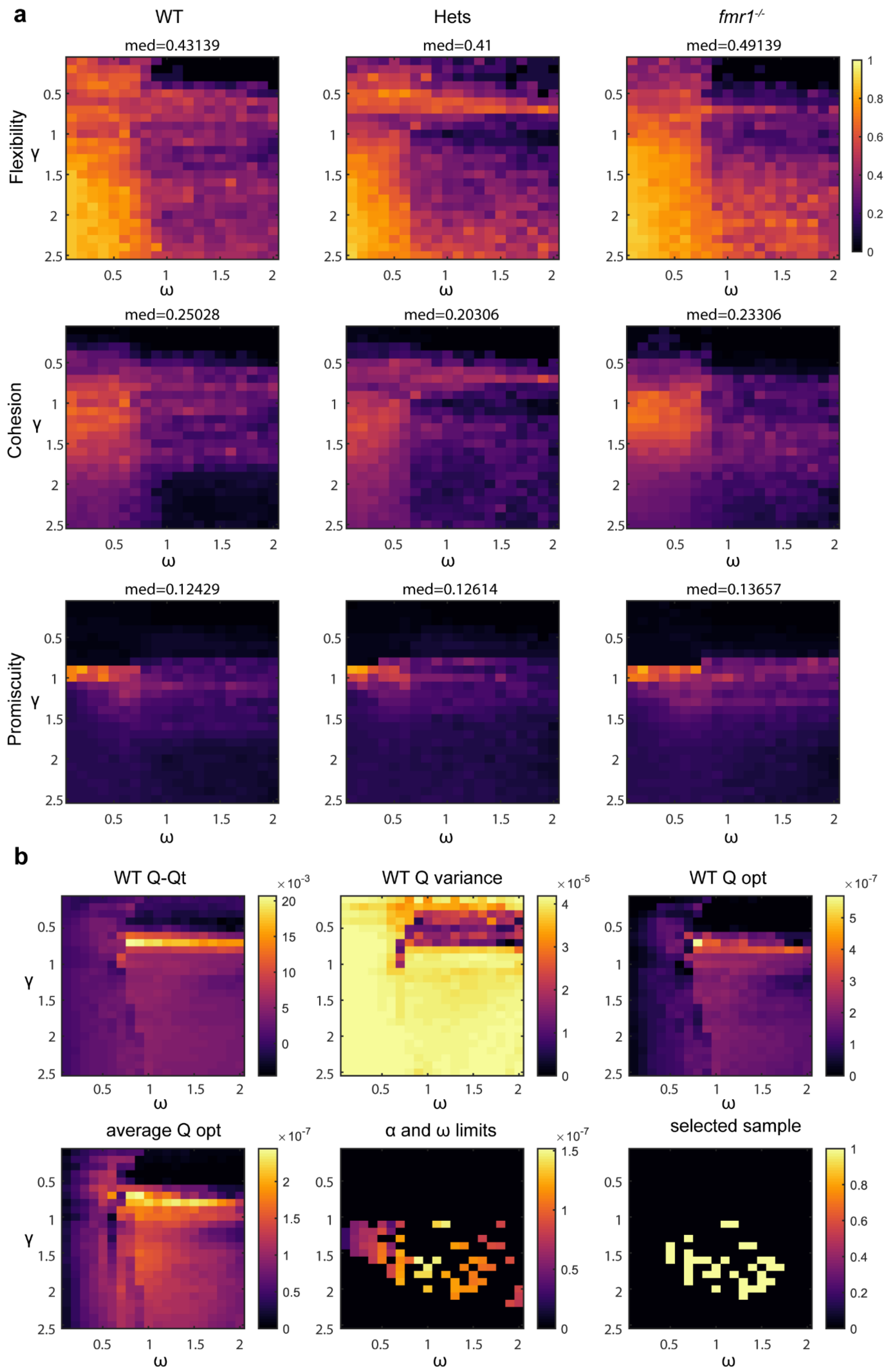

a
WT
Hets
fmr1-/-
med=0.43139
med=0.41
med=0.49139
Flexibility
γ
ω
med=0.25028
med=0.20306
med=0.23306
Cohesion
med=0.12429
med=0.12614
med=0.13657
Promiscuity
b
WT Q-Qt
WT Q variance
WT Q opt
average Q opt
α and ω limits
selected sample

Supplementary Figure 10. Dynamic community measures and optimization of γ and ω parameters.

a. Range of the average flexibility, cohesion, and promiscuity results based on combinations of the γ and ω parameters for the multilayer graphs of the WT, Hets, and *fmr1* mutants. The median is given on the top of each subfigure. **b.** Steps for selecting the γ and ω values. Top left, difference in Q (maximized modularity quality, see Methods) of the WT graph and the temporal null model of the WT graph at various γ and ω values. Top middle, relative variance of the maximum Q of the WT graph. Higher values represent the combinations with lowest variance. Top right, optimization of Q by combining the Q-Qt difference and the relative variance of the WT graphs. Higher values indicate combinations of parameters that have a larger difference from the temporal null model and also show low variance. Bottom left, average of the optimized Q of the 3 genotype datasets; optimized Q is defined as the mean value of the maximized Q that have the highest difference from the null model and the lowest variance. Bottom middle, same as bottom left but applying the limiting rules for community detection results (See Methods). Bottom right, combination of values that are above the mean in the bottom middle panel. This set of parameters was used to estimate the community measures reported in Figure 6.

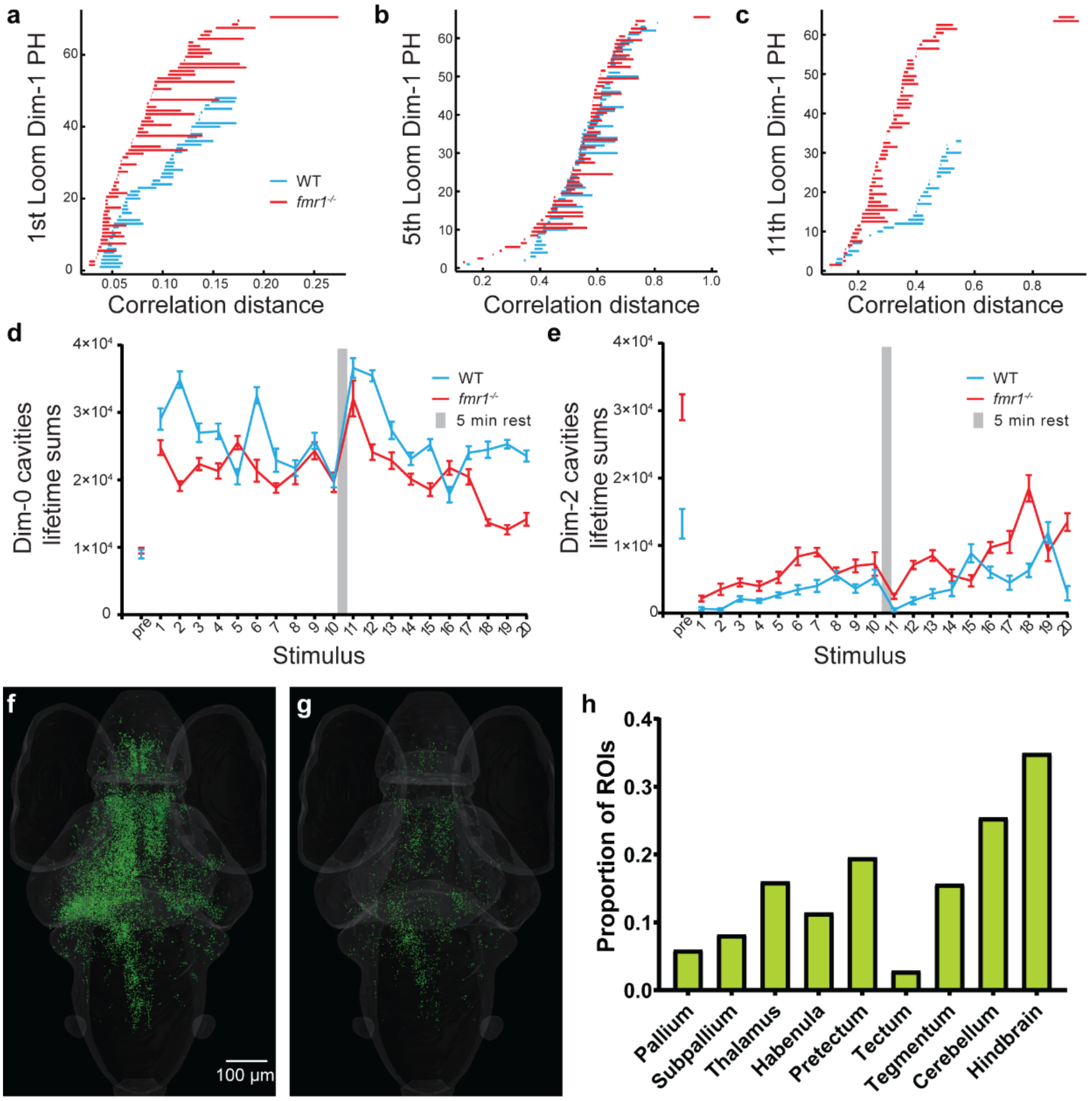

Supplementary Figure 11. Persistent homology analysis and motor related strongly habituating ROIs.

a-e. Barcode graphs and lifetime sums of the persistent homology analysis performed in leave-one out group-averaged matrices (see Methods, WT=10 and *fmr1*$^{-/-}$=11). **a.** Dimension 1 barcode graphs for *fmr1* mutants at the 1st loom. **b.** Dimension 1 barcode graphs for *fmr1* mutants and WT at the 5th loom. **c.** Dimension 1 barcode graphs for *fmr1* mutants and WT at the 11th loom. **d.** Dimension 0 lifetime sums of group-averaged matrices of *fmr1* mutants (n=11) and WT (n=10) at pre-loom and 20 loom time points. **e.** Dimension 2 lifetime sums of group-averaged matrices of *fmr1* mutants (n=11) and WT (n=10) at pre-loom and 20 loom time points. Centre indicate means and error bars indicate 95% CIs in **e** and **d**.

f-h. Prominence of motor-correlated strongly habituating ROIs in the hindbrain. **f.** The distribution of all strongly habituating neurons across the brain for the f20 stimulus train. **g.** The subset of the ROIs from panel **(f)** that show >1 s.d. correlation with motor responses during loom stimuli on a trial-by-trial basis. **h.** The proportion of strongly habituating ROIs that shows this motor correlation, by brain region.

Supplementary Table 1. Analysis of proportion of ROIs in each region (Figure 3b)

	Mean Diff.	Tukey's multiple comparisons test 95.00% CI of diff.	Summary	Individual P Value	Adjusted P Value
**Pallium**					
Strong Hab vs. Moderately Hab	0.02723	-0.02387 to 0.07833	ns	0.2070	0.415
Strong Hab vs. Weakly Hab	-0.06476	-0.1159 to -0.01366	**	0.0033	0.0092
Moderately Hab vs. Weakly Hab	-0.09199	-0.1431 to -0.04089	***	<0.0001	0.0001
**Subpallium**					
Strong Hab vs. Moderately Hab	0.02144	-0.02966 to 0.07254	ns	0.3195	0.5779
Strong Hab vs. Weakly Hab	0.0197	-0.03140 to 0.07080	ns	0.3601	0.629
Moderately Hab vs. Weakly Hab	-0.00174	-0.05284 to 0.04936	ns	0.9354	0.9964
**Thalamus**					
Strong Hab vs. Moderately Hab	0.1034	0.05226 to 0.1545	****	<0.0001	<0.0001
Strong Hab vs. Weakly Hab	0.1006	0.04949 to 0.1517	****	<0.0001	<0.0001
Moderately Hab vs. Weakly Hab	-0.00277	-0.05387 to 0.04833	ns	0.8974	0.9908
**Habenula**					
Strong Hab vs. Moderately Hab	0.000388	-0.05071 to 0.05149	ns	0.9856	0.9998
Strong Hab vs. Weakly Hab	-0.1579	-0.2090 to -0.1068	****	<0.0001	<0.0001
Moderately Hab vs. Weakly Hab	-0.1583	-0.2094 to -0.1072	****	<0.0001	<0.0001
**Pretectum**					
Strong Hab vs. Moderately Hab	0.003205	-0.04789 to 0.05431	ns	0.8813	0.9877
Strong Hab vs. Weakly Hab	-0.06029	-0.1114 to -0.009194	*	0.0061	0.0166
Moderately Hab vs. Weakly Hab	-0.0635	-0.1146 to -0.01240	*	0.0040	0.0109
**Tectum**					
Strong Hab vs. Moderately Hab	-0.466	-0.5171 to -0.4149	****	<0.0001	<0.0001
Strong Hab vs. Weakly Hab	-0.09744	-0.1485 to -0.04634	****	<0.0001	<0.0001
Moderately Hab vs. Weakly Hab	0.3686	0.3175 to 0.4197	****	<0.0001	<0.0001
**Tegmentum**					
Strong Hab vs. Moderately Hab	0.04854	-0.002556 to 0.09964	ns	0.0260	0.0662
Strong Hab vs. Weakly Hab	0.06273	0.01163 to 0.1138	*	0.0044	0.0121
Moderately Hab vs. Weakly Hab	0.01418	-0.03692 to 0.06528	ns	0.5094	0.7857
**Hindbrain**					
Strong Hab vs. Moderately Hab	0.1242	0.07308 to 0.1753	****	<0.0001	<0.0001
Strong Hab vs. Weakly Hab	0.1248	0.07369 to 0.1759	****	<0.0001	<0.0001
Moderately Hab vs. Weakly Hab	0.000612	-0.05049 to 0.05171	ns	0.9772	0.9995

Supplementary Table 1. Analysis of proportion of ROIs in each region
Two-way ANOVA test (one-sided) performed to compare the differences in cluster proportion of ROIs across brain regions in the f20, f60, s20 and s60 datasets.

Supplementary Table 2. Analysis of cluster identity of loom-responsive ROIs (Figure 3c)

	Tukey's multiple comparisons test				
	Mean Diff.	95.00% CI of diff.	Summary	Individual P Value	Adjusted P Value
**Pallium**					
Strong Hab vs. Moderately Hab	0.4001	0.2904 to 0.5098	****	<0.0001	<0.0001
Strong Hab vs. Weakly Hab	0.1353	0.02561 to 0.2450	*	0.0042	0.0116
Moderately Hab vs. Weakly Hab	-0.2648	-0.3745 to -0.1551	****	<0.0001	<0.0001
**Subpallium**					
Strong Hab vs. Moderately Hab	0.7912	0.6815 to 0.9009	****	<0.0001	<0.0001
Strong Hab vs. Weakly Hab	0.7689	0.6592 to 0.8786	****	<0.0001	<0.0001
Moderately Hab vs. Weakly Hab	-0.02225	-0.1320 to 0.08745	ns	0.6295	0.8789
**Thalamus**					
Strong Hab vs. Moderately Hab	0.6455	0.5358 to 0.7552	****	<0.0001	<0.0001
Strong Hab vs. Weakly Hab	0.6206	0.5109 to 0.7303	****	<0.0001	<0.0001
Moderately Hab vs. Weakly Hab	-0.02489	-0.1346 to 0.08482	ns	0.5896	0.851
**Habenula**					
Strong Hab vs. Moderately Hab	0.09772	-0.01199 to 0.2074	ns	0.0365	0.0908
Strong Hab vs. Weakly Hab	-0.4966	-0.6063 to -0.3869	****	<0.0001	<0.0001
Moderately Hab vs. Weakly Hab	-0.5943	-0.7040 to -0.4846	****	<0.0001	<0.0001
**Pretectum**					
Strong Hab vs. Moderately Hab	0.2911	0.1814 to 0.4008	****	<0.0001	<0.0001
Strong Hab vs. Weakly Hab	0.06758	-0.04212 to 0.1773	ns	0.1452	0.3103
Moderately Hab vs. Weakly Hab	-0.2235	-0.3332 to -0.1138	****	<0.0001	<0.0001
**Tectum**					
Strong Hab vs. Moderately Hab	-0.1133	-0.2231 to -0.003647	*	0.0157	0.0412
Strong Hab vs. Weakly Hab	0.2334	0.1236 to 0.3431	****	<0.0001	<0.0001
Moderately Hab vs. Weakly Hab	0.3467	0.2370 to 0.4564	****	<0.0001	<0.0001
**Tegmentum**					
Strong Hab vs. Moderately Hab	0.6031	0.4934 to 0.7128	****	<0.0001	<0.0001
Strong Hab vs. Weakly Hab	0.6873	0.5776 to 0.7970	****	<0.0001	<0.0001
Moderately Hab vs. Weakly Hab	0.08417	-0.02553 to 0.1939	ns	0.0706	0.1657
**Hindbrain**					
Strong Hab vs. Moderately Hab	0.6348	0.5251 to 0.7445	****	<0.0001	<0.0001
Strong Hab vs. Weakly Hab	0.6447	0.5350 to 0.7544	****	<0.0001	<0.0001
Moderately Hab vs. Weakly Hab	0.009862	-0.09984 to 0.1196	ns	0.8306	0.9749

Supplementary Table 2. Analysis of cluster identity of loom-responsive ROIs
Two-way ANOVA test (one-sided) performed to compare the differences in cluster identity within brain regions across the f20, f60, s20 and s60 datasets.

Supplementary Table 3. Relative difference analysis of dynamic community measures

		Friedman's test		Multiple comparisons			
	Median diff.	$\chi^2_{2=}$	p value	Mean rank diff.	95% CI L	95% CI U	p value*
Flexibility							
Pallium	-0.0025	12.41176	0.002018	-0.38235	-0.96298	0.198272	0.344745
Subpallium	-0.05625	16.28148	0.000291	-0.94118	-1.51966	-0.36269	**0.000295**
Thalamus	-0.03958	29.17647	4.62E-07	-0.47059	-1.05121	0.110037	0.157035
Habenula	-0.05	24.76471	4.19E-06	-0.64706	-1.22768	-0.06643	0.022899
Pretectum	-0.1325	37.64444	6.69E-09	-0.97059	-1.54908	-0.3921	**0.000177**
Tectum	-0.11447	38.35294	4.70E-09	-1	-1.58063	-0.41937	**0.000112**
Tegmentum	-0.16042	40.41176	1.68E-09	-1.05882	-1.63945	-0.4782	**3.80E-05**
Cerebellum	-0.15	27.44361	1.10E-06	-0.73529	-1.30948	-0.16111	0.006515
Hindbrain	-0.24333	45.70588	1.19E-10	-1.14706	-1.72768	-0.56643	**6.75E-06**
Strongly habituating	-0.14821	41.82353	8.28E-10	-1.14706	-1.72768	-0.56643	**6.75E-06**
Moderately habituating	0	18.58824	9.20E-05	-0.23529	-0.81592	0.345331	0.995926
Weakly habituating	-0.08235	25.11765	3.51E-06	-0.47059	-1.05121	0.110037	0.157035
Inhibited	-0.125	29.83077	3.33E-07	-0.69118	-1.25885	-0.1235	0.010677
Cohesion							
Pallium	-0.0425	9.588235	0.008278	-0.5	-1.08063	0.080625	0.117751
Subpallium	-0.0625	16.16418	0.000309	-0.83824	-1.41458	-0.2619	**0.001494**
Thalamus	-0.03542	31.11765	1.75E-07	-0.67647	-1.2571	-0.09585	0.015854
Habenula	0.145	9.644444	8.05E-03	0.75	0.171513	1.328487	0.005732
Pretectum	-0.075	17.17037	1.87E-04	-0.98529	-1.56378	-0.40681	**0.000137**
Tectum	0.043421	3.941176	1.39E-01	-0.32353	-0.90415	0.257096	0.546665
Tegmentum	-0.1125	34.58824	3.09E-08	-0.88235	-1.46298	-0.30173	**8.24E-04**
Cerebellum	-0.075	2.396947	3.02E-01	-0.36765	-0.9375	0.202205	0.367398
Hindbrain	-0.07167	31.29412	1.60E-07	-0.58824	-1.16886	-0.00761	4.59E-02
Strongly habituating	-0.12411	37.82353	6.12E-09	-1.17647	-1.7571	-0.59585	**3.69E-06**
Moderately habituating	0.013333	6.711111	3.49E-02	-0.39706	-0.97555	0.181428	0.301045
Weakly habituating	0.227941	15.94118	3.45E-04	0.852941	0.272316	1.433567	**0.001311**
Inhibited	-0.125	29.83077	3.33E-07	-0.69118	-1.25885	-0.1235	0.010677
Promiscuity							
Pallium	0.019648	2.503704	0.285975	0.323529	-0.25496	0.902016	0.541832
Subpallium	0.002024	6.977778	0.030535	0.044118	-0.53437	0.622604	1
Thalamus	0.032615	4.941176	0.084535	0.352941	-0.22768	0.933567	0.43683
Habenula	0.006271	9.941176	0.006939	-0.02941	-0.61004	0.551214	1
Pretectum	-0.00632	15.23529	0.000492	-0.67647	-1.2571	-0.09585	0.015854
Tectum	0.011417	11.70588	0.002871	-0.32353	-0.90415	0.257096	0.546665
Tegmentum	-0.0117	12.76471	0.001691	-0.55882	-1.13945	0.021802	0.063653
Cerebellum	-0.02991	7.268657	0.026402	-0.36765	-0.94399	0.208693	0.380195
Hindbrain	-0.04557	53.11765	2.92E-12	-1.32353	-1.90415	-0.7429	**1.45E-07**
Strongly habituating	-0.00882	22.29412	1.44E-05	-0.85294	-1.43357	-0.27232	**0.001311**
Moderately habituating	0.014194	16.64706	0.000243	0.205882	-0.37474	0.786508	1
Weakly habituating	0.020789	12.76471	0.001691	-0.02941	-0.61004	0.551214	1
Inhibited	-0.01633	17.73333	0.000141	-0.57353	-1.15202	0.004957	0.052866

* p value adjusted for multiple comparisons of the three groups (WT, Hets and *fmr1* mutants) with the Bonferroni method.
Bold indicates p values bellow a Bonferroni correction for multiple tests (0.05/13= 0.0038).

Supplementary Table 3. Relative difference analysis of dynamic community measures

Friedman's test (one-sided) performed to compare the differences in Flexibility, Cohesion and Promiscuity between WT and *fmr1* mutants.

Supplementary Movie 1.

Anatomical locations for the ROIs belonging to each functional cluster. 3D rotation of Figure 2h.
