# Supplementary material for: Brain-wide visual habituation networks in wild type and fmr1 zebrafish
Source: Nat Commun. 2022 Feb 16;13:895. doi: 10.1038/s41467-022-28299-4 (PMC8850451; doi:10.1038/s41467-022-28299-4)
Supplement: Supplementary file 2 — Reporting Summary [file 41467_2022_28299_MOESM2_ESM.pdf]

nature portfolio

Corresponding author(s): Ethan Scott

Last updated by author(s): Dec 6, 2021

Reporting Summary

Nature Portfolio wishes to improve the reproducibility of the work that we publish. This form provides structure for consistency and transparency in reporting. For further information on Nature Portfolio policies, see our Editorial Policies and the Editorial Policy Checklist.

Statistics

For all statistical analyses, confirm that the following items are present in the figure legend, table legend, main text, or Methods section.

n/a	Confirmed	
☐	☒	The exact sample size (*n*) for each experimental group/condition, given as a discrete number and unit of measurement
☐	☒	A statement on whether measurements were taken from distinct samples or whether the same sample was measured repeatedly
☐	☒	The statistical test(s) used AND whether they are one- or two-sided<br>*Only common tests should be described solely by name; describe more complex techniques in the Methods section.*
☐	☒	A description of all covariates tested
☐	☒	A description of any assumptions or corrections, such as tests of normality and adjustment for multiple comparisons
☐	☒	A full description of the statistical parameters including central tendency (e.g. means) or other basic estimates (e.g. regression coefficient) AND variation (e.g. standard deviation) or associated estimates of uncertainty (e.g. confidence intervals)
☐	☒	For null hypothesis testing, the test statistic (e.g. *F*, *t*, *r*) with confidence intervals, effect sizes, degrees of freedom and *P* value noted<br>*Give P values as exact values whenever suitable.*
☒	☐	For Bayesian analysis, information on the choice of priors and Markov chain Monte Carlo settings
☐	☒	For hierarchical and complex designs, identification of the appropriate level for tests and full reporting of outcomes
☐	☒	Estimates of effect sizes (e.g. Cohen's *d*, Pearson's *r*), indicating how they were calculated

Our web collection on statistics for biologists contains articles on many of the points above.

Software and code

Policy information about availability of computer code

Data collection	Pylon viewer v5.2.0 (Basler); Basler Video Recording Software v1.3 (Basler); Viewpoint software v4.4 (ZebraLab, ViewPoint Life Sciences, France); Micro-<br>Manager v1.4.22; ImageJ v1.52c
Data analysis	We used Fiji (v1.52c, Java 1.8.0_172) to make hyperstacks of the .tif files acquired with uManager (v1.4.22) and then split in z planes time series. We then used CaImAn version 0.9 (http://github.com/flatironinstitute/CaImAn) to analyze the images and perform a functional segmentation to extract the calcium traces of each ROI from each z plane. We used 4000 components per slice to ensure that we would not miss any ROIs during the initialization step of CaImAn. The risk of over-segmentation was mitigated by a merge step using a threshold of 0.8 to merge overlapping ROIs. The order of the autoregressive model was set at 1 to account for the decay of the fluorescence, our acquisition speed being too slow to account for the rise time. The gSig (half-size of neurons) was set at 2, based on estimates of the sizes of the nuclei in our images. We did not use any temporal or spatial down-sampling and the initialization method was 'greedy_roi'.<br>We used Advanced Normalization Tools (ANTs, https://github.com/ANTsX/ANTs) to compute the diffomorphic map between time-averaged 3D image stacks of each fish and the H2B-RFP reference of Z-brain (https://zebrafishexplorer.zib.de/home/). The same mapping was used to warp the centroid coordinates for each ROI of interest to the H2B-RFP reference, which includes 294 segmented brain regions.<br>The active ROIs and their calcium traces were then analyzed using custom-written code in Matlab R2018b.<br><br>matlab functions used:<br><br>For graph analysis we used the Brain Connectivity Toolbox for matlab: https://www.mathworks.com/matlabcentral/fileexchange/61173-brain-connectivity-toolbox<br><br>AAFT function from: Barnett, A. Test of non-linearity (https://www.mathworks.com/matlabcentral/fileexchange/16062-test-of-non-linearity), MATLAB Central File Exchange. Retrieved December 11, 2019. (2019).

The multilayer graphs and dynamic community detection is based on previous work 79 and was performed on the unthresholded matrices using the MATLAB genlouvain.m function from the GenLouvain v2.2 toolbox.

The colormaps used for Figures 2, 4-7 and Supplementary Figures 2, 4-8 were generated using two Matlab® functions: The cbrewer function, https://au.mathworks.com/matlabcentral/fileexchange/34087-cbrewer-colorbrewer-schemes-for-matlab (Accessed in May 2019) which includes specifications and designs developed by Cynthia Brewer (http://colorbrewer.org/), and the MatPlotLib 2.0 default colormaps ported to Matlab, https://au.mathworks.com/matlabcentral/fileexchange/62729-matplotlib-2-0-colormaps-perceptually-uniform-and-beautiful (Accessed in May 2019).

The circular graphs (Figure 6 and Supplementary Figure 9) were made with a modified version of the code from Matlab®'s circularGraph toolbox. https://www.mathworks.com/matlabcentral/fileexchange/48576-circulargraph/ (Accessed in May 2019).

The density plot in Supplementary Figure 4b was made with dscatter function. Made by Robert Henson and found in Flow Cytometry Data Reader and Visualization (https://www.mathworks.com/matlabcentral/fileexchange/8430-flow-cytometry-data-reader-and-visualization), MATLAB Central File Exchange. (Accessed in November 2020).

Other software used to analyze the data were: GraphPad Prism v8.3.1; RStudio 1.1.456; R 3.5.1 and R 3.6.2; Julia 0.6.4; Unity v2019.3.0a2.

Scripts for the analysis can be found in the github repository: https://github.com/emarquezUQ/ZF_Loom_Habituation_MarquezLegorreta_et_al_2021/releases/tag/v1.0.0.

All data are stored at the University of Queensland Research Data Manager repository, and will also be made available upon request. The .tif files, processed variables and scripts generated in this study have been deposited in the "MarquezLegorreta_et_al_2021_Datasets" database with the identifier https://doi.org/10.48610/9549fdc. Source data is provided with this paper.

For manuscripts utilizing custom algorithms or software that are central to the research but not yet described in published literature, software must be made available to editors and reviewers. We strongly encourage code deposition in a community repository (e.g. GitHub). See the Nature Portfolio guidelines for submitting code & software for further information.

Data

Policy information about availability of data

All manuscripts must include a data availability statement. This statement should provide the following information, where applicable:

- Accession codes, unique identifiers, or web links for publicly available datasets
- A description of any restrictions on data availability
- For clinical datasets or third party data, please ensure that the statement adheres to our policy

All data are stored at the University of Queensland Research Data Manager repository, and will also be made available upon request. The .tif files, processed variables and scripts generated in this study have been deposited in the "MarquezLegorreta_et_al_2021_Datasets" database with the identifier https://doi.org/10.48610/9549fdc. Source data is provided with this paper.

Field-specific reporting

Please select the one below that is the best fit for your research. If you are not sure, read the appropriate sections before making your selection.

☒ Life sciences ☐ Behavioural & social sciences ☐ Ecological, evolutionary & environmental sciences

For a reference copy of the document with all sections, see nature.com/documents/nr-reporting-summary-flat.pdf

Life sciences study design

All studies must disclose on these points even when the disclosure is negative.

Sample size	No sample size calculation was performed. Sample size is reported for each experiment throughout the document. The free-swimming behavioral experiment suggested that general behavioral trends could be detected already with a sample size of 12 animals (each clutch tested). Furthermore, due to the amount of information and size of the data, it is common in the field to use a sample of 5-10 animals for whole-brain calcium imaging experiments. Therefore, for our experiments, our aim was to acquire a minimum of 5 animals per group, but trying to get closer to 10 animals.
Data exclusions	As stated in the text, 3 fish (1 from f20 and 2 from f60) were discarded because their contribution to one of the habituating clusters was above 50% of the total number of ROIs for that cluster, so they were deemed as outliers in terms of responsiveness. The threshold for this criterion was not pre-established, but we had as a prior criterion that the data should reflect responses from all or the majority of fish in a relative proportional manner.<br>For behavioral experiments, fish could be excluded if they didn't present any startle response, they were not traceable, the clutch was considered unhealthy or there was an error with the stimulus delivery.
Replication	No explicit reproduction was carried out. However, the data was drawn from experiments that were performed with fish from multiple clutches and on multiple days, which indicates that the results are reproducible. Furthermore, experiments conducted afterwards for the fmr1 part of the manuscript show very similar general results (type of responses and distribution of ROIs in the brain). This also corroborates that the main results can be replicated.

Randomization	Animals were allocated to the four stimulus trains at random.
Blinding	fmr1 experiments were inherently blind, as genotyping took place after the collection of data and quantification. For the other experiments the experimenter was not blinded. We consider that being blinded when performing the experiments was not relevant as the results were analyzed together once all the groups reached the desired sample size (n=~10). Blinding for the analysis was not practically feasible as the profile of responses had clear differences between groups (e.g. length, shape).

Reporting for specific materials, systems and methods

We require information from authors about some types of materials, experimental systems and methods used in many studies. Here, indicate whether each material, system or method listed is relevant to your study. If you are not sure if a list item applies to your research, read the appropriate section before selecting a response.

Materials & experimental systems

n/a	Involved in the study
☒	☐ Antibodies
☒	☐ Eukaryotic cell lines
☒	☐ Palaeontology and archaeology
☐	☒ Animals and other organisms
☒	☐ Human research participants
☒	☐ Clinical data
☒	☐ Dual use research of concern

Methods

n/a	Involved in the study
☒	☐ ChIP-seq
☒	☐ Flow cytometry
☒	☐ MRI-based neuroimaging

Animals and other organisms

Policy information about studies involving animals; ARRIVE guidelines recommended for reporting animal research

Laboratory animals	Danio rerio of the TLN strain. Adults (>90 dpf) of both sexes were used for breeding of the larvae used in experiments. 6dpf larvae of undetermined sex (sex not differentiated until 21-23 dpf) were use for the free-swimming and calcium imaging experiments.
Wild animals	No wild animals were used in this study.
Field-collected samples	No field-collected samples were used in this study.
Ethics oversight	The University of Queensland Animal Welfare Unit.

Note that full information on the approval of the study protocol must also be provided in the manuscript.
