# Supplementary material for: Brain-wide visual habituation networks in wild type and fmr1 zebrafish
Source: Nat Commun. 2022 Feb 16;13:895. doi: 10.1038/s41467-022-28299-4 (PMC8850451; doi:10.1038/s41467-022-28299-4)
Supplement: Supplementary file 4 — Description of Additional Supplementary Files [file 41467_2022_28299_MOESM4_ESM.pdf]

Title: Supplementary Movie 1

Description: Anatomical locations for the ROIs belonging to each functional cluster. 3D rotation of Figure 2h.
